# Low Levels of Peripheral CD161++CD8+ Mucosal Associated Invariant T (MAIT) Cells Are Found in HIV and HIV/TB Co-Infection

**DOI:** 10.1371/journal.pone.0083474

**Published:** 2013-12-31

**Authors:** Emily B. Wong, Ngomu Akeem Akilimali, Pamla Govender, Zuri A. Sullivan, Cormac Cosgrove, Mona Pillay, David M. Lewinsohn, William R. Bishai, Bruce D. Walker, Thumbi Ndung'u, Paul Klenerman, Victoria O. Kasprowicz

**Affiliations:** 1 KwaZulu-Natal Research Institute for Tuberculosis and HIV, Durban, South Africa; 2 Division of Infectious Diseases, Massachusetts General Hospital, Boston, Massachusetts, United States of America; 3 HIV Pathogenesis Programme, University of KwaZulu-Natal, Durban, South Africa; 4 Division of Infectious Diseases, Johns Hopkins School of Medicine, Baltimore, Maryland, United States of America; 5 Peter Medawar Building for Pathogen Research, University of Oxford, Oxford, United Kingdom; 6 Division of Pulmonary and Critical Care Medicine, Oregon Health & Science University, Portland, Oregon, United States of America; 7 Portland Veterans Administration Medical Center, Portland, Oregon, United States of America; 8 The Ragon Institute of MGH, MIT, and Harvard, Harvard Medical School, Cambridge, Massachusetts, United States of America; 9 Max Planck Institute for Infection Biology, Berlin, Germany; University of Cape Town, South Africa

## Abstract

**Background:**

High expression of CD161 on CD8+ T cells is associated with a population of cells thought to play a role in mucosal immunity. We wished to investigate this subset in an HIV and *Mycobacterium tuberculosis* (MTB) endemic African setting.

**Methods:**

A flow cytometric approach was used to assess the frequency and phenotype of CD161++CD8+ T cells. 80 individuals were recruited for cross-sectional analysis: controls (n = 13), latent MTB infection (LTBI) only (n = 14), pulmonary tuberculosis (TB) only (n = 9), HIV only (n = 16), HIV and LTBI co-infection (n = 13) and HIV and TB co-infection (n = 15). The impact of acute HIV infection was assessed in 5 individuals recruited within 3 weeks of infection. The frequency of CD161++CD8+ T cells was assessed prior to and during antiretroviral therapy (ART) in 14 HIV-positive patients.

**Results:**

CD161++CD8+ T cells expressed high levels of the HIV co-receptor CCR5, the tissue-homing marker CCR6, and the Mucosal-Associated Invariant T (MAIT) cell TCR Vα7.2. Acute and chronic HIV were associated with lower frequencies of CD161++CD8+ T cells, which did not correlate with CD4 count or HIV viral load. ART was not associated with an increase in CD161++CD8+ T cell frequency. There was a trend towards lower levels of CD161++CD8+ T cells in HIV-negative individuals with active and latent TB. In those co-infected with HIV and TB, CD161++CD8+ T cells were found at low levels similar to those seen in HIV mono-infection.

**Conclusions:**

The frequencies and phenotype of CD161++CD8+ T cells in this South African cohort are comparable to those published in European and US cohorts. Low-levels of this population were associated with acute and chronic HIV infection. Lower levels of the tissue-trophic CD161++ CD8+ T cell population may contribute to weakened mucosal immune defense, making HIV-infected subjects more susceptible to pulmonary and gastrointestinal infections and detrimentally impacting on host defense against TB.

## Introduction

CD161++ CD8+ T cells have recently been brought to the forefront of research on the cellular immune response to a number of infectious diseases [1] [2], [3]. Northfield et al reported that CD161 expression indicates a unique pattern of CD8+ T cell differentiation, tightly linked to co-expression of CXCR6 (a chemokine receptor with a major role in liver homing)[1]. Other studies reported two sub-populations of CD161 cells based on staining intensity [2]. The CD161++CD8+ T cell population, which expresses high levels of the C-type lectin CD161 on the cell surface, is noted for its ability to produce IL-17A and IL-22 (in addition to TNF-alpha and IFN-gamma), cytokines important in the maintenance of mucosal integrity and antibacterial immunity [2], [4]–[7]. In addition this population, that expresses tissue-homing markers including CCR6 and CXCR6, is enriched in tissue samples including the liver and the joints [2]. More recently, an important overlap between expression of CD161 and the antimicrobial Mucosal Associated Invariant T cells (MAITs) has been reported with up to 80–90% of CD161++ cells co-expressing a semi-invariant T cell receptor that features Vα7.2 [8]–[10]. MAITs recognize bacterial riboflavin metabolite ligands presented by the MHC-related protein 1 (MR1) and are activated by pathogens including *Escherichia coli*, *Candida albicans* and *Mycobacterium tuberculosis* (MTB) [10], [11]). Importantly, these cells have been shown to provide protection against bacterial infection [12], [13]. For example, Chua et al demonstrated that MAIT cells inhibited bacterial growth of *Bacillus Calmette–Guérin* (BCG) in macrophages in an MR1-dependent manner [12]. This group also reported higher bacterial loads in MAIT-cell deficient mice infected with BCG compared to wild-type mice, highlighting their significant role in anti-bacterial immunity [12]. Reports from humans from our group and others indicate that this cell population is depleted from the blood of tuberculosis (TB) patients [13]–[15].

HIV and MTB mono and co-infection are significant and interconnected problems in Africa. Between 1990 and 2005, the TB incidence rate in Africa was estimated to have increased by an annual average of 6%, driven predominantly by the HIV epidemic [16]. South Africa has the greatest burden of HIV infected individuals with at least 5.7 million infected people [17]. It is estimated that HIV has led to a 3-fold increase in TB prevalence in South Africa over the last decade, and the country now has the second highest estimated TB incidence per capita, with TB being the leading cause of death [18], [19]. The province of KwaZulu-Natal (KZN) is the epicenter of the HIV and TB co-epidemic in South Africa. KZN has an estimated 1.2 million HIV positive individuals, an antenatal HIV prevalence of 37.4%, a TB notification rate of 12,900/100,000 population, and an HIV co-infection rate of 70–80% of TB cases [20], [21]. A striking feature of these two infections is that there is an increased risk of MTB infection and progression to TB in HIV infected individuals, even before their immune system is compromised to levels at which other opportunistic infections occur [22]. In addition, even on effective ART, this TB vulnerability is not fully repaired; suggesting that loss of the CD4+ T cell population is not the sole mechanism responsible[22], [23].

Recent data from two groups have indicated that CD161++/MAIT cells are lost from the blood in patients infected with HIV [9], [24], [25]. Although both studies identified a very clear impact of HIV on circulating CD161++ frequencies, they were discrepant on the relationship with CD4 count and the impact of co-infections was not assessed. Therefore, while loss of CD161++/MAIT cells could contribute to the excess risk of TB in early-stage HIV infection, further examination is required, especially in a setting where the risk is very high.

We therefore wished to further investigate the CD161++ CD8+ T cell population in the context of HIV and MTB mono and co-infection. As this population is present in cord blood and thought to expand post-natally in response to the gut microbiome, regional differences in frequency and phenotype of this population may exist [11], [26]–[28]. We therefore investigated how the CD161++ CD8+ T cell subset is affected by infection with HIV and/or MTB in KZN, South Africa, where HIV and TB are devastating co-epidemics.

## Methods

### Patients

Ethical approval was obtained form the University of KwaZulu-Natal Biomedical Research Ethics Committee (BFC 115/09 ‘Characterization of *M.tb*-specific immunity in HIV infected and uninfected South Africa Adults’) and written informed consent was obtained from all patients.

A total of 80 individuals in six different states of HIV and MTB mono- and co-infection were recruited for cross-sectional analysis in KwaZulu-Natal, South Africa: Healthy subjects (HIV negative and TB negative, HNTN (n = 13), HIV negative with latent TB infection (HNLTBI) (n = 14), HIV negative with active tuberculosis (TB) (HNTP) (n = 9), HIV positive TB negative (HPTN, n = 16), HIV positive with LTBI (HPLTBI, n = 13) and HIV positive with active TB (HPTP, n = 15) (Table 1). All patients were participants in the iThimba cohort based in KZN, South Africa. All patients were ARV and TB therapy naïve at baseline. All active TB subjects were pulmonary TB cases identified by a positive sputum AFB or sputum culture. All individuals defined as having LTBI were asymptomatic, had a positive ESAT-6 and/or CFP-10 (RD1)-specific IFN-gamma ELISPOT result, and were smear and culture negative for MTB on induced sputum[29]. All individuals defined as negative for TB were asymptomatic, RD-1 IFN-gamma ELISPOT negative and sputum smear and culture negative for MTB on induced sputum.

**Table 1:** Clinical and demographic characteristics of the individuals whose samples were used to assess the frequency and phenotype of the CD161++ CD8+ T cell population.

Group	Number	Age	Female Sex (%)	CD4 cell count/μl	HIV Viral Load copies/ml
HNTN	13	36 (22–55)	77	803 (479–1772)	
HNLTBI	14	37 (26–47)	86	1056 (591–1196)	
HNTP	9	42 (18–55)	33	775 (668–1196)	
HPTN	16	37 (27–54)	81	493 (248–940)	30,959 (856–588,160)
HPLTBI	13	31 (25–51)	85	388 (231–1325)	21,721 (20–211,623)
HPTP	15	29 (22–47)	60	291 (13–949)	66,000 (198–650,000)

HNTN: HIV negative, tuberculosis (TB) negative; HNLTBI: HIV negative, latent tuberculosis infection (LTBI)); HNTP: HIV negative, TB positive; HPTN: HIV positive, TB negative; HPLTBI: HIV positive, LTBI; HPTP: HIV positive, TB positive. Except where designated, all values are expressed as median (range).

In addition, we recruited 5 individuals in the acute stage of HIV infection (within 2–3 weeks of presumed infection date) who were assessed regularly (for a maximum of 23 weeks (as detailed in [30]). To assess the impact of ART, the frequency of CD161++CD8+ cells was assessed prior to and during ART in 14 HIV-positive patients (median duration of treatment was 172 days (range = 92–282), all patients suppressed their HIV viral load to undetectable by the time of the “on ART” measurement) (Table 2).

**Table 2:** Clinical and demographic characteristics of the individuals whose samples were used to assess the impact of antiretroviral therapy (ART) on the frequency of the CD161++ CD8+ T cell population.

Group	Number	Age	Female Sex (%)	Pre-ART CD4 cell count/μl	Pre-ART HIV viral load/ml	Post-ART CD4 cell count/μl	Post-ART HIV viral load/ml	Days on ART
HNTN	14	37 (20–53)	93	935.5 (544–1772)	N/A	N/A	N/A	N/A
HPTN	14	33 (20–51)	80	310 (211–425)	34,000 (350–420,000)	352.5 (262–775)	0 (0–98)	172 (92–282)

HNTN: HIV negative, tuberculosis (TB) negative; HPTN: HIV positive, TB negative. Except where designated, all values are expressed as median (range).

### Flow cytometric studies

All antibodies were pre-titrated to determine appropriate working concentrations. For the phenotypic characterization of CD161++ CD8+ T cells, whole blood was stained directly and the erythrocytes subsequently lysed with Fix/VersaLyse (Beckman Coulter) and stained with the following antibodies: CD3 Pacific orange (Invitrogen, clone UCHT1), CD4 Qdot 605 (Invitrogen, clone S3.5), CD8 V450 (BD Bioscience, clone RPA-T8) and CD161 APC (Mitenyi Biotech, clone 191B8). Surface staining for specific receptors was performed using monoclonal antibodies directed against: CD103 FITC (clone Ber-ACT8), CCR6 PerCP Cy5.5 (clone 11A9), CXCR4 PE Cy-7 (clone 12G5), or CCR5 PE (clone 2D7/CCR5, all antibodies from BD Biosciences). Additional analyses (including staining for Vα7.2 expression and longitudinal assessment of response to ART) were performed on cryopreserved PBMC using the following monoclonal antibodies: CD3 Alexafluor 700 (BD, clone UCHT1), CD8 APC-H7 (BD, clone SK1) CD4 PerCP Cy5.5 (Biolegend, clone RPA-T4), CD161 PECy7 (Biolegend, clone HP-3G10), Vα7.2 APC (courtesy of Gold laboratory, OHSU), CD14 PE (Biolegend). Surface stains were incubated for 20 minutes at 4C (PBMC) and room temperature (whole blood). Cells were analyzed on an LSRII cytometer using FACSDiva software. Data were analyzed using FloJo (v9.3.1 and v10). Doublets were excluded based on FSC-H and FSC-A, lymphocytes were identified based on FSC and SSC, and dead cells were excluded based on Infra-Red or Aqua viability dye (Invitrogen). CD3+ and CD8+ lymphocytes were identified. Fluorescence minus one (FMO) controls were used for optimal gating.

### Statistics

Two-sided Mann-Whitney tests for non-parametric data were used to compare groups. Threshold for significance was 0.05 with adjustment using the Bonferroni correction in cases of multiple comparisons. Pairwise longitudinal comparisons were performed using Wilcoxon matched pairs signed rank test. Correlations between CD161++ CD8+ T cell frequency and markers of HIV disease progression were assessed using Spearman's rank.

## Results

### CD161++CD8+ T cells display a distinct phenotype in healthy individuals from KwaZulu-Natal

**Figure 1:**
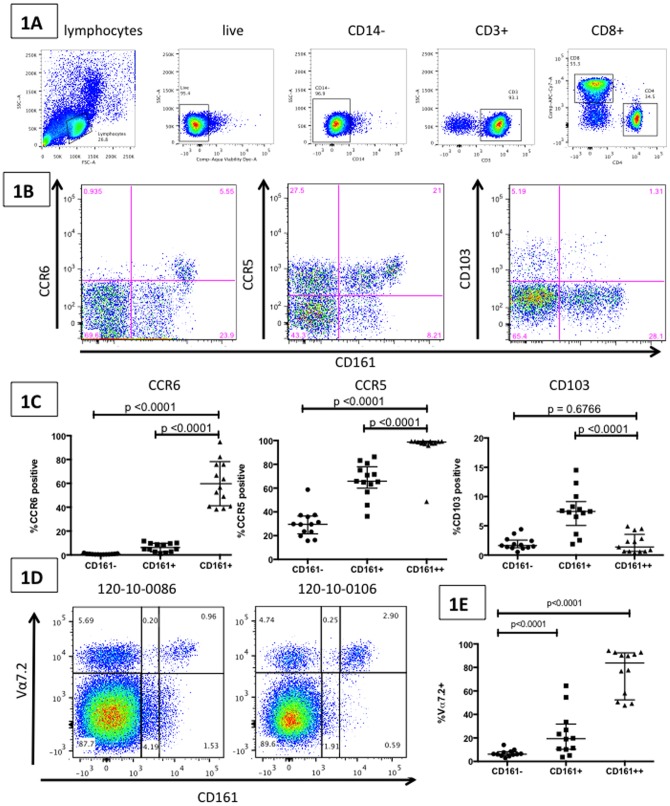
CD161++ CD8+ MAIT cells have a distinct phenotypic profile. Figure 1A: Depiction of the gating strategy used throughout: lymphocytes were selected on the basis of forward- and side-scatter characteristics, dead cells excluded, then CD14 negative, CD3 positive, CD4 negative, CD8 positive cells selected. Figure 1B: FACS plots (gated on the CD8+ T cell population) show CD161++ cells demonstrating high expression of CCR6 and CCR5 and low expression of CD103 in a representative example from an HIV negative TB negative subject (HNTN). Figure 1C: Aggregate data from 13 HNTN individuals demonstrate that CD161++ CD8+ T cells express significantly higher levels of CCR5 and CCR6 and lower levels of CD103 compared to CD161+ and CD161- CD8+ T cells. Figure 1D: FACS plots (gated on the CD8+ T cell population) show high expression of the Vα7.2 semi-invariant T-cell receptor by CD161++ T cells from two representative HNTN individuals. Figure 1E: Aggregate data from 12 HNTN individuals demonstrate that CD161++CD8+ T cells express significantly higher levels of the Vα7.2 TCR than either CD161+CD8+ T cells or CD161-CD8+ T cells. All graphs show median and intra-quartile range; p-values are reported for two-sided Mann-Whitney tests with threshold for significance p = 0.025 after Bonferroni correction for 2 comparisons.

In 13 healthy individuals (HIV negative, TB negative), CD161++CD8+ T cells were observed to express high levels of the tissue-homing markers CXCR4, CCR5 and CCR6, and low-level expression of CD103 (liver/gut homing marker) [31], [32]. Significantly higher expression of CCR5 (median CCR5 expression on CD161++ CD8+ T cells = 98.7%), and CCR6 (median CCR6 expression on CD161++ CD8+ T cells = 56.6%) were observed on CD161++ CD8+ T cells compared to CD161+ CD8+ T cells (CCR5: p<0.0001 and CCR6: p<0.0001) and CD161- CD8+ T cells (CCR5: p<0.0001 and CCR6: p<0.0001) (Fig. 1B, Fig. 1C). Significantly lower levels of expression of CD103 were observed on CD161++ CD8+ T cells (median CD103 expression on CD161++ CD8+ T cells = 1.38%) compared to CD161+ CD8+ T cells (median CD103 expression on CD161+ CD8+ T cells = 7.46%, p<0.0001), similar to expression levels observed on CD161- CD8+ T cells (Fig. 1C). CXCR4 was expressed at high levels in CD161++, CD161+ and CD161- CD8+ T cells, and expression levels were not able to differentiate between the different groups (data not shown). In another subset of healthy subjects, a large proportion of CD161++ CD8+ T cells were observed to co-express the semi-invariant T cell receptor Vα7.2 (median = 83.8%, range: 47.7–92.9), a marker of the anti-bacterial Mucosal Associated Invariant T cells (MAITs). This compared to a median of 19.4% (p<0.001) for CD161+ CD8+ T cells and 6.2% of CD161- CD8+ T cells (p<0.001) (Fig. 1D and Fig. 1E). Overall these data are consistent with previous studies of this cell subset performed in healthy individuals from the US and Europe [2], [8], [9], [24], [26], [33].

### HIV and TB are associated with low levels of anti-microbial CD161++ CD8+ T cells

**Figure 2:**
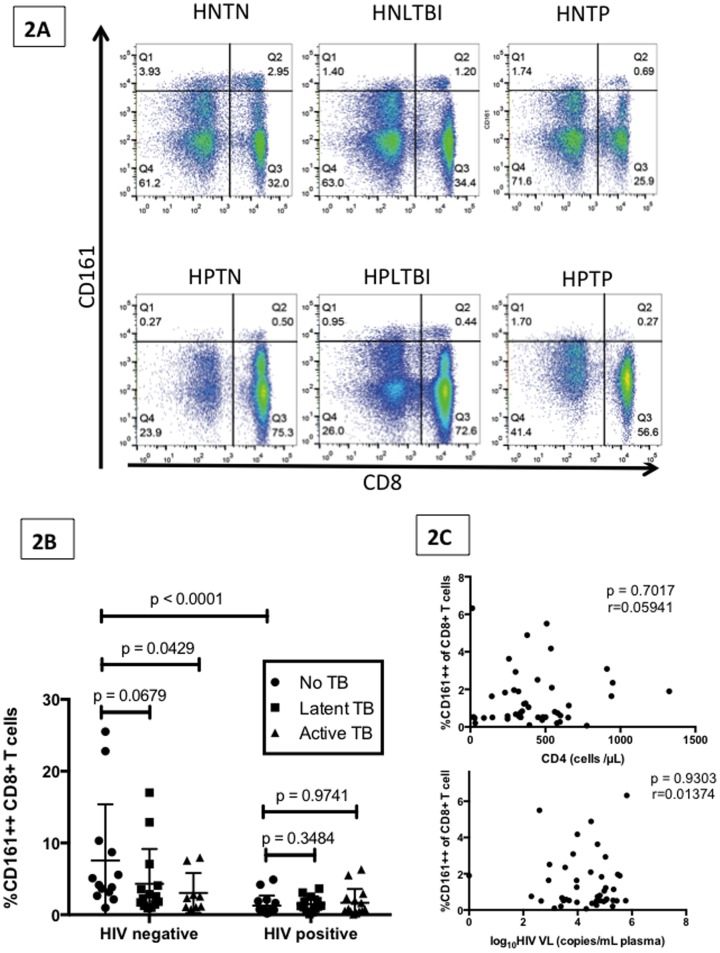
Impact of HIV and TB mono- and co-infection on CD161++ CD8+ T cell frequencies. Figure 2A: FACS plots (gated on the CD3+ T cell population) show CD161 and CD8 co-staining on representative individuals from 6 different clinical groups: HIV negative and TB negative (HNTN), HIV negative with latent TB infection (HNLTBI), HIV negative with active TB (HNTP), HIV positive TB negative (HPTN), HIV positive with latent TB infection (HPLTBI) and HIV positive with active TB (HPTP). Figure 2B: Aggregate data demonstrating CD161++ CD8+ T cell frequency (median and interquartile range) in 6 different groups: HNTN (n = 13), HNLTBI (n = 14), HNTP (n = 9), HPTN (n = 16), HPLTBI (n = 13) HPTP (n = 15). P-values reported for two-sided Mann-Whitney tests, with a p-value threshold for significance of 0.01 after Bonferroni correction for 5 comparisons. Figure 2C: CD161++CD8+ T cell frequency in all subjects with HIV-infection showed no significant correlation with either CD4 count (cells/μl) or HIV viral load (copies/ml).

We investigated the effects of HIV and MTB mono- and co-infection on the CD161++CD8+ T cell frequency using the 6 groups described above (their demographic and clinical characteristics are summarized in Table 1). In ‘healthy’ (HNTN) individuals the median CD161++CD8+ T cell frequency was 4.14% (range 0.96–25.5) of all CD8+ T cells (Fig. 2B). HIV mono-infection (HPTN) was associated with significantly lower levels of CD161++ CD8+ T cells compared to healthy individuals (median = 0.59%, range = 0.19–4.89; p<0.0001). Among HIV-negative subjects, individuals with active TB infection demonstrated a trend towards lower CD161++ CD8+ T cell frequency (median = 2.33%, range = 0.75–7.97; p = 0.0429). In HIV-negative subjects, those with latent TB infection also demonstrated a trend towards lower CD161++ CD8+ T cell frequency (median = 2.41%, range = 0.92–17.0; p = 0.0679). The above observations were not seen for the CD161+CD8+ T cell frequency, which did not show changes across the different clinical groups (data not shown). Among those subjects with HIV, TB co-infection (either active TB or LTBI) did not further lower CD161++CD8+ T cell population compared to those with HIV mono-infection (median = 0.75%, range = 0.07–6.3; p = 0.97 and median = 1.22%, range = 0.095–3.63; p = 0.35 respectively, Fig. 2B).

CD161++CD8+ T cell frequency in HIV positive individuals did not correlate with either CD4 count (cells/μL) or HIV viral load (copies/mL) (figure 2C). Interestingly, both TB and HIV mono-infection were associated with statistically significant lower-level expression of the mucosal-homing receptor CCR6 on the CD161++ CD8+ T cell population compared to that observed in healthy individuals (median CCR6 expression in healthy subjects was 56.6%, in HIV mono-infection 37.3%; p = 0.0125, and in TB mono-infection 30.2%; p = 0.0284). All other phenotypic markers remained stable, including CCR5 (data not shown).

### Acute HIV infection is associated with lower frequencies of CD161++ CD8+ T cells

**Figure 3:**
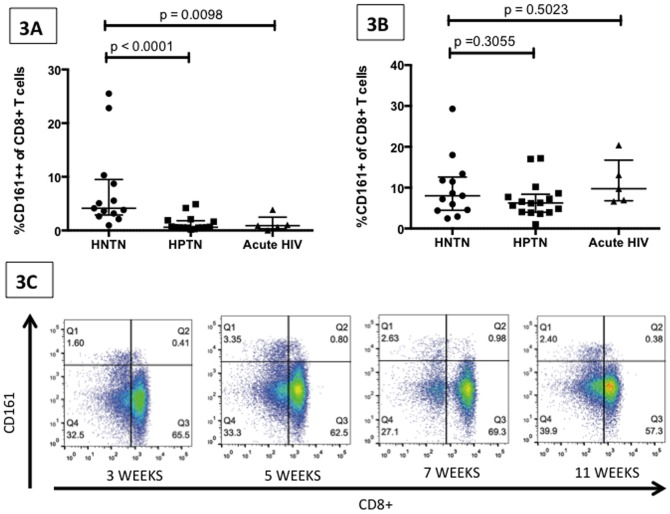
Acute HIV infection is associated with a lower frequency of CD161++ CD8+ T cells. Figure 3A: Graph displays CD161++ CD8+ T cell frequency in HIV negative and TB negative subjects (HNTN, n =  13), individuals with chronic HIV infection (HIV positive TB negative HPTN, n = 16), and individuals in the acute phase of HIV infection (n = 5, sample taken within 2–3 weeks of presumed infection date). CD161++CD8+ T cells are significantly lower in HPTN individuals and those with acute HIV. Figure 3B: Graph displays CD161+CD8+ T cell frequency in the same three groups of subjects; CD161+ CD8+ T cells do not show any differences between these three groups. Figure 3C: A representative FACS plot displaying the CD161++ CD8+ T cell population over time in an individual acutely infected with HIV and assessed at 5 timepoints. Both graphs show median and interquartile range; p-values are reported for two-sided Mann-Whitney tests with threshold for significance p = 0.025 after Bonferroni correction for 2 comparisons.

CD161++CD8+ T cells were measured in five subjects with acute HIV who were observed very early in acute infection (first observed 15–21 days post presumed date of infection, 20% female, median age 36 years). Significantly lower levels of CD161++ CD8+ T cells (comparable to those seen in chronic HIV infection) were found in these individuals in the acute stage of HIV infection compared to healthy controls (HNTN) (median = 0.900%, range = 0.03–3.89; p = 0.0098; Fig. 3A). Similar decreases in the CD161+CD8+ T cell population were not observed (Fig. 3B). When acutely HIV-infected patients were followed longitudinally over the first 24 weeks post-infection, small fluctuations in the CD161++CD8+ population were observed but population percentages remained within the low range observed in those with chronic HIV infection (Fig. 3C shows one characteristic example).

### Impact of ART on CD161++/MAIT cell frequency

**Figure 4:**
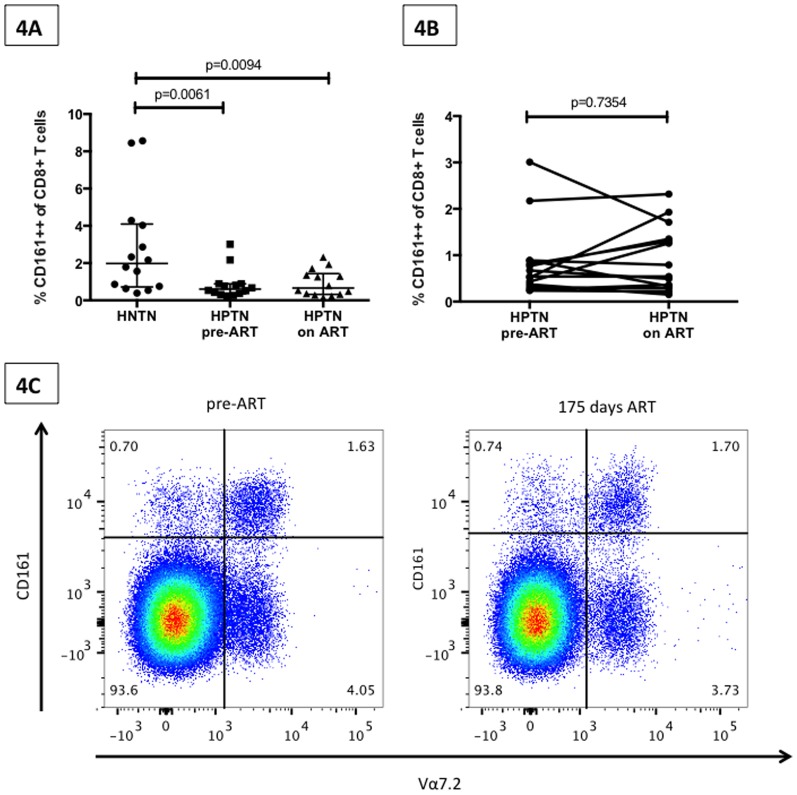
Impact of Antiretroviral treatment (ART) on CD161++ CD8+ T cell frequency. Figure 4A: Graph displaying CD161++CD8+ T cell frequency (median and interquartile range) from 14 HIV negative TB negative individuals (HNTN) and 14 HIV positive TB negative individuals (HPTN) prior to ART initiation (pre-ART) and on virally suppressive ART (on ART). P-value reported from two-sided Mann-Whitney tests with threshold for significance p = 0.025 after Bonferroni correction for 2 comparisons. Figure 4B: Paired CD161++CD8+ T-cell frequencies in HIV-positive individuals prior to and following virally suppressive ART (p-value reported for Wilcoxon matched pairs test). Figure 4C: FACS plots (gated on the CD8+ T cell population) displaying the lack of change in the CD161++ CD8+ population (co-stained with the MAIT cell semi-invariant TCR, Vα7.2) prior to and after 6 months of ART in a representative subject.

We next investigated the impact of ART on our population of interest, using fourteen subjects from the iThimba cohort who initiated ART and achieved viral suppression during the period of observation. The demographic and clinical description of these subjects is summarized in Table 2. As we were using cryopreserved PBMC we first determined if the pattern of lower CD161++ CD8+ T cell frequency in HIV infected (untreated) compared to healthy individuals was confirmed in this sample type. Figure 4A shows that we again saw significantly lower levels of the CD161++ CD8+ T cell population in HIV infected individuals compared to healthy controls (p = 0.0095). ART that effectively suppressed HIV viral load (median duration 172 days, range 92–282) was not associated with an increase in CD161++ CD8+ T cell frequency (pre-ART median = 0.665, on ART median = 0.605; p = 0.7354) (Fig. 4B). The median fold change in CD161++ CD8+ T cell frequency was 0.97 (range = 0.37–3.71). Fig. 4C shows representative FACS plots of the CD161++ CD8+ population (co-stained for Vα7.2) prior to and after approximately 6 months of ART.

## Discussion

In our group of individuals based in Durban, South Africa we identified a population of CD8+ T cells that expressed very high levels of the C-type lectin CD161. This population (between 1-26% of CD8+ T cells in healthy (HIV and TB negative) individuals) had a distinct phenotypic profile and expressed distinctly high levels of the HIV co-receptor CCR5 and the tissue-homing marker CCR6. In addition, the vast majority of this population expressed the Vα7.2 TCR, consistent with a MAIT cell population. The CD161++ CD8+ T cell frequency and phenotype observed in our cohort is in line with recent reports in populations based in Europe and the US ([2], [8], [9], [24], [26]. This CD161++/MAIT cell subset may play a significant role in antimicrobial immunity in both HIV and MTB infections. This is the first study to investigate this CD161++/MAIT cell population in HIV infected, MTB infected (both LTBI and TB), and co-infected individuals in an HIV- and TB-endemic setting.

Chronic HIV infection was associated with significantly lower levels of CD161++ CD8+ T cells. This finding is supported by recent reports by Cosgrove et al and Leeansyah et al [9], [24]. Interestingly, in all of the data sets there are healthy individuals who have low CD161++ CD8+ T cell frequencies, in the same range as individuals with HIV. This raises the question of whether low-levels of this cell subset make individuals more susceptible to HIV infection, or if HIV infected individuals undergo a decrease of this subset in the bloodstream. Cosgrove et al investigated the frequency of CD161++ CD8+ T cells in early HIV infection (baseline sample was taken within 6 months of HIV infection), reporting that the frequency of CD161++ CD8+ T cells was already reduced in this phase of infection [9]. Our data from individuals in the acute phase of HIV infection reveal that low-level frequencies of this MAIT cell population are present even 2-3 weeks post presumed HIV infection date, indicating either that a rapid change occurs, or that these low levels were present in these individuals prior to infection. To clarify this issue, this question needs to be addressed with a larger population of individuals, with both pre- and post-infection time-points. The CD161++/MAIT cell subset did not show further declines in frequency in longitudinal follow-up of our acutely infected population (up to 24 weeks post-presumed infection date). Interestingly, this is in contrast to Leeansyah et al, who reported that the decline of MAIT cells (defined as CD161+, Vα7.2+, and primarily CD8+) in HIV infected individuals is associated with time since diagnosis [24].

It is unlikely that the ‘reduced frequencies’ of this MAIT cell population is merely a result of dilution due to expansion of HIV-specific subsets following infection as virus-specific CD8+ T cells do not contribute significantly to the CD161++ CD8+ T cell pool (data not shown and [9]) and MAIT cells are activated by a range of bacteria and fungi (but not viruses) in a MR1-dependent manner. In addition the low frequency of this population in HIV-infected subjects is only observed for CD161++ CD8+ T cells and not CD161+ CD8+ T cells, which appear to be stably maintained in HIV infected individuals. Cosgrove et al provide *in vitro* support for the possibility that the CD161++ CD8+ T cell population is depleted by activation-induced apoptosis on encounter with translocated bacteria [9]. Other potential scenarios are that this cell population is redistributed to tissues upon infection, which could explain in part the low levels of CCR6 expression on this subset in HIV and TB infected individuals. This area requires further work as to date only one report has emerged from the literature, which found no enrichment of this population in colon tissue from HIV infected individuals [9]. We investigated if ART was able to increase the observed frequency of the CD161++ CD8+ MAIT cell population and did not find a significant change following up to 282 days of treatment (a finding supported by [9], [24]). Interestingly, in our study the CD161++ CD8+ T cell frequency did not correlate with CD4 count or HIV viral load in HIV positive individuals.

Individuals with active pulmonary TB displayed a trend towards lower levels of CD161++CD8+ T cells compared to ‘healthy’ individuals, however the significance of the finding (p = 0.043) did not survive the stringency of a 5 comparison Bonferroni correction. Since our finding supports previous reports that the MAIT cell population is depleted from the blood of tuberculosis (TB) patients, it is likely that the borderline significance is the result of our relatively small sample sizes [13]–[15]. Further investigation on a larger cohort needs to be performed, especially to dissect the impact of LTBI and subclinical infection. Interestingly, HIV-TB co-infection was not associated with a synergistic decrease in frequency when compared to HIV mono-infected individuals. It will be interesting to monitor the CD161++CD8+ MAIT cell population in individuals who progress from LTBI to active disease and those who undergo TB. Further work in tissues also needs to be performed.

In conclusion, low levels of CD161++ CD8+ MAIT cells in peripheral blood samples were associated with HIV infection and HIV/TB co-infection. Interestingly, low levels are found in acute HIV infection (2–3 weeks post presumed infection) suggesting that either low frequencies of this population predispose individuals to HIV infection, or that this population of cells is either destroyed as a consequence of infection or is redistributed to the site of infection. ART was unable to increase the frequency of this MAIT cell population, however TB therapy in HIV uninfected individuals may offer this possibility. Lower levels of the tissue-homing CD161++ CD8+ T cell population may contribute to a weakened mucosal immune defense in subjects infected with HIV and TB, making HIV subjects more susceptible to diseases like TB, and detrimentally impacting on the host's battle with TB.
