# Correction: Low Levels of Peripheral CD161++CD8+ Mucosal Associated Invariant T (MAIT) Cells Are Found in HIV and HIV/TB Co-Infection

**DOI:** 10.1371/journal.pone.0095115

**Published:** 2014-04-08

**Authors:** The PLOS ONE Staff

## Notice of Republication

This article was republished on March 21, 2014, to replace an incorrectly published version. The publisher apologizes for the error. Please download this article again to view the correct version. The originally published, uncorrected article and the republished, corrected article are provided here for reference.

## Supporting Information

File S1 Originally published, uncorrected article.
(PDF)

File S2 Republished, corrected article.
(PDF)
